# Urban Scaling Functions: Emission, Pollution and Health

**DOI:** 10.1007/s11524-024-00888-2

**Published:** 2024-07-12

**Authors:** Caterina A. M. La Porta, Stefano Zapperi

**Affiliations:** 1 Center for Complexity and Biosystems and Center for Innovation for Well-Being And Environment, Department of Environmental Science and Policy, University of Milan, via Celoria 10, Milan, 20133 Italy; 2 UOC Maxillo-Facial Surgery and Dentistry, Fondazione IRCCS Cá Granda, Ospedale Maggiore Policlinico di Milano, via Francesco Sforza, 28, Milan, 20122 Italy; 3 Center for Complexity and Biosystems, Department of Physics “Aldo Pontremoli”, University of Milan, via Celoria 16, Milan, 20133 Italy; 4 Istituto di Chimica della Materia Condensata e di Tecnologie per l’Energia, Consiglio Nazionale delle Ricerche, via R. Cozzi 53, Milan, 201 Italy

**Keywords:** Urban scaling, Air pollution, Carbon emission, Respiratory diseases

## Abstract

Urban scaling is widely used to characterize the population dependence of city indicators including greenhouse gas emission. Here we analyze the population dependence of CO_2_ and PM2.5 emissions and concentrations across all European cities. Our analysis reveals considerable variations in emissions among cities of comparable population size which are not captured by conventional urban scaling. We thus characterize these fluctuations by multi-parameter scaling functions and multifractal spectral analysis. We find that the distribution of emissions and population is multifractal while that of air pollution is not, leading to non-trivial relations between emission and pollution in some large cities. We also analyze the impact of forests in curbing emission and the impact of air pollution on health. Our work provides a detailed picture of the fluctuations in the scaling of urban metabolism in Europe and suggests a general strategy that goes beyond conventional urban scaling laws.

**Supplementary Information:**

The online version contains supplementary material available at 10.1007/s11524-024-00888-2.

## Introduction

Urban settlements are an important source of global anthropogenic greenhouse gases and air pollutants, currently accounting for a third of total on a global scale [1, 2]. Given the growing urbanization trends, understanding how city size affects emissions and air pollution is a pressing question that has spurred considerable investigation and debate in last decade [3–6]. Considering that each individual bears direct or indirect responsibility for a specific level of CO_2_ emissions, the question arises as to whether large urban areas contribute to more or less emissions per capita when compared to less populated areas. This question can be reframed through the lens of urban scaling laws which draw inspiration from allometric laws employed in the description of biological organisms. Empirical evidence shows that the metabolic rate and other biologically relevant quantities exhibit a power law dependence on the typical body size of the organism [7–9]. The functioning of cities has been described in terms of urban metabolism [10], reflecting the flow and transformation of resources within a city and all the interconnected processes that sustain urban life. It was thus reasonable to assume that allometric scaling could be used to describe the quantitative relations between relevant indicators of urban metabolism and the population size [11–16].

Population scaling in greenhouse gas emissions is usually verified by plotting the total CO_2_ emitted by the city $E_{\textrm{CO2}}$ against its population size *P* in a double logarithmic plot [3–6, 17]. If emissions depend on the population size as $E_{\textrm{CO2}} \sim P^\beta$ with $\beta < 1$, then emissions per capita ($E_{\textrm{CO2}}/P$) are smaller in larger cities. On the other hand if $\beta >1$, then concentrating the population in larger and larger cities would be detrimental for emissions. While the question is very well posed, the answer is still debated since estimates of the exponent $\beta$ have been found to be either close to one [3], larger than one [4] or smaller than one [6]. These discrepancies have been attributed to the data used, to the definition of city boundaries [4] or to the method used to estimate the exponents [5].

The release of greenhouse gases and air pollutants has dual consequences: a worldwide impact on the environment by contributing to global warming, and a local effect manifested in the deterioration of air quality within urban areas. Fine particulate matter (PM2.5) is of particular concern due to its detrimental effects on health: prolonged exposure to high PM2.5 concentrations is considered a risk factor for respiratory infections [18, 19] and lung cancer [20]. Previous studies on the relation between the concentration of PM2.5 in the air and urban population size, however, reported conflicting results either showing power law scaling [21] or non-monotonic relationships [22].

Here we address the population scaling of emission and pollution in European cities by focusing on the fluctuations around the mean. We propose scaling functions describing the joint distribution of emission and population and verify the scaling assumptions using emission and pollution data from various sources [1, 23–25]. We also consider the geometric variability of the emission scaling in terms of a multifractal analysis, following earlier studies suggesting that population represents a non-trivial multifractal measure [26, 27]. Finally, we discuss the relevance of mitigation strategies based on carbon capture by forests [28] and the health impact of PM2.5 air pollution in Europe.

## Methods

### Data

Shapefiles and populations sizes for local administrative units (LAU) and Nomenclature of territorial units for statistics (NUTS) were downloaded from the GISCO EUROSTAT website (https://ec.europa.eu/eurostat/web/gisco/) for the year 2018. PM2.5 emission data where extracted from the EDGAR database (v6.1) [23] for the year 2018 (https://edgar.jrc.ec.europa.eu/dataset_ap61 ). CO_2_ emissions for the year 2018 were extracted from the EDGAR database (v8.0) [24] for the year 2018 (https://edgar.jrc.ec.europa.eu/dataset_ghg80). Concentrations for PM2.5 at grid level were downloaded from the European Environmental Agency (EEA) (https://www.eea.europa.eu/en/datahub/ file n. 938bea70-07fc-47e9-8559-8a09f7f92494) for the year 2018. CO_2_ emission data from OpenGHGMap [25] were downloaded from https://openghgmap.net/data/. European mountains shapefiles were obtained from the European Environment agency (https://www.eea.europa.eu/en/datahub/ file n. 48b6f8a7-9e5a-4865-bd05-59c29348b5fb). Forest land cover was extracted from EUROSTAT Land cover for FAO Forest categories by NUTS 2 regions https://ec.europa.eu/eurostat/web/lucas/database. CO_2_ captured by forests for European countries was obtained from the Annual European Union greenhouse gas inventory 1990-2019 and inventory report 2021 [29] table 6.8. Mortality data were extracted from the EUROSTAT, Causes of death - standardised death rate by NUTS 2 region of residence, website https://ec.europa.eu/eurostat/databrowser/view/hlth_cd_ysdr2/.

### Statistical Analysis of Geolocalized Data

Geolocalized data analysis was performed in python importing the data using geopandas https://geopandas.org/. Interpolation of emission and concentration data on city areas specified by LAU shapefiles was performed using the tobler package https://github.com/pysal/tobler. We estimate conditional expectation values $\langle X(Y) \rangle$ of a first variable *X* (e.g., CO_2_ emission) with respect to a second variable *Y* (e.g., population size), defined as the mean of *X* when the second variable is equal to *Y*. In practice, we use logarithmicaly spaced bins by computing the logarithm of the variable ($Z=\log _{10} Y$), defining a set of linearly spaced intervals and calculating the averages of *X* for all the points for which *Z* falls into the interval considered. A similar procedure is followed to estimate conditional distributions $\rho (X|Y)$.

### Multifractal Analysis

To perform the multifractal scaling analysis [30] the variable of interest (population, CO_2_ and PM2.5 emission, PM2.5 concentration) is considered as a measure $\mu$. We then aggregate the measure in grids with square cells with side *b* and evaluate the partition function for different values of *b* defined as

1 $$\begin{aligned} Z_q(b) = \sum _i \mu _b(i)^q \end{aligned}$$

where $\mu _b(i)$ is the measure of interest (i.e., *P*, $E_{CO2}$, $E_{PM2.5}$, or CO_2_) aggregated over the cell *i* of linear size *b* and *q* is the moment value, chosen in the [0, 5] interval. The multifractal exponents $\tau (q)$ are obtained from the scaling of the partition function with the cell size $Z_q(b) \sim b^{\tau (q)}$. The multifractal spectrum is then obtained from the Lagrange transform $f(\alpha )=q\alpha -\tau (q)$ where $\alpha =d\tau /dq$ implemented using finite differences. The python package GeoMF used to perform the analysis is available at https://github.com/ComplexityBiosystems/GeoMF.

### Health Impact Assessment

To compute PAF for the disease of interest, we used data from the Global Burden of Disease (GBD) Study 2019 [31] https://ghdx.healthdata.org/ providing the empirical relation between exposure to PM2.5 and the PAF for lung cancer, COPD and ALRI. We use the local concentration of PM2.5 in each region to estimate the PAF from the GBD relation.

## Results

### Distributions of CO_2_ and PM2.5 Emissions from Cities Scale with Population Size

**Fig. 1:**
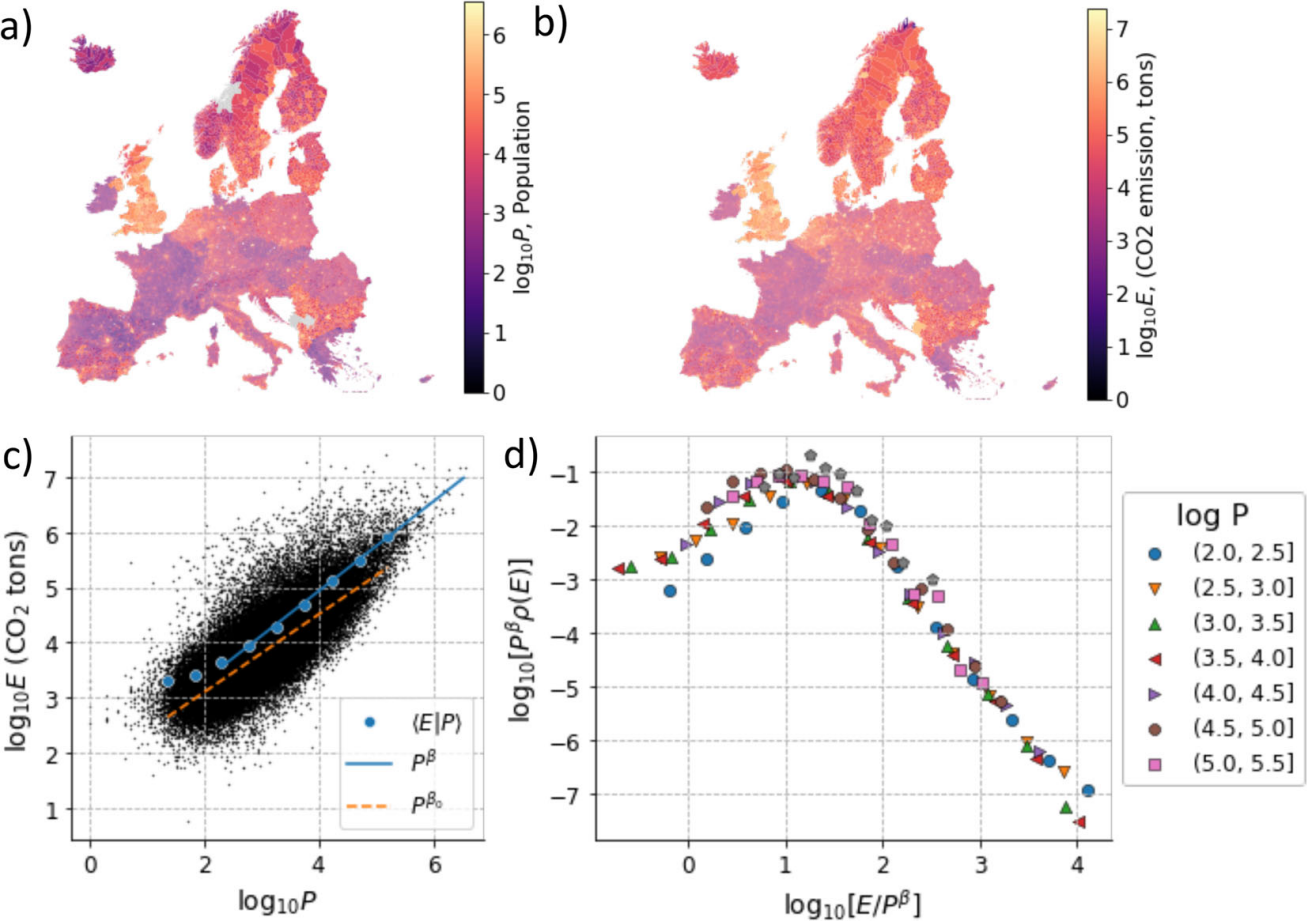
Scaling of CO_2_ emission with urban population. (a) Map of the population values of European urban areas. (b) Map of CO_2_ emissions (as logarithm of the number of tons) of European urban areas. (c) Relation between the logarithms of CO_2_ emissions (*E* CO_2_) and population (*P*), including the expectation value $\langle E|P \rangle$ ithe fit with $P^\beta$, with $\beta =0.81$. Notice that the fit is performed only over the linear part of the curve. We also report the power law fit over all the data, yielding $\beta _0=0.7$. (d) Data collapse of the conditional distributions of CO_2_ emissions at fixed population using $\beta =0.81$. Data from GISCO-EUROSTAT and EDGAR (v8.0) for the year 2018

To study the scaling of urban CO_2_ and PM2.5 emissions with population in Europe, we consider the Edgar emission database [23, 24] and aggregate it over Local Administrative Units (LAU), using the population size for each city reported in the LAU data. We consider data relative to the year 2018 for which data is available for both CO_2_ and PM2.5 emissions. Population sizes and CO_2_ emissions are broadly distributed and highly heterogeneous across Europe as illustrated by mapping the $\log$ of population *P* and CO_2_ emission $E_{\textrm{CO2}}$ (Fig. 1a and b) and by inspecting the distributions of population size and CO_2_ emissions of European cities (Fig. S1). The relation between CO_2_ emission *E* and population size *P* is plotted in Fig. 1c using a double logarithmic scale. We also plot the conditional expectation value of the CO_2_ emission for a given population size $\langle E_{\textrm{CO2}}|P\rangle$ which grows as a power law $P^\beta$, with $\beta =0.81$. This value is slightly different from the value obtained by fitting all the data ($\beta _0=0.7$). The difference is due to the large fluctuations in emissions observed among cities with similar population sizes.

A better characterization of the relation between CO_2_ emissions and population sizes can be obtained by considering multi-parameter scaling functions. In particular, we evaluate the conditional distribution of CO_2_ emissions for a given population size $\rho (E_{\textrm{CO2}}|P)$ and assume that it obeys a scaling function

2 $$\begin{aligned} \rho (E_{\textrm{CO2}}|P) = P^{-\beta } \mathcal {F}(E_{\textrm{CO2}} P^{-\beta }). \end{aligned}$$

The simple form of the scaling function is dictated by normalization of the conditional distribution as can be shown as follows. Consider a generic scaling function for the conditional distribution of two variables x and y, $\rho (x|y)= y^{-a} f(xy^{-b})$. Normalization implies that $\int _0^\infty \rho (x|y)dx= \int _0^\infty y^{-a} f(xy^{-b}) dx = 1$. Changing variables as $z=xy^{-b}$, we obtain $y^{b-a}\int _0^\infty f(z) dx = 1$ which can be only be satisfied if $a=b$.

Rescaling the measured conditional distributions according to Eq. 2 with $\beta =0.81$ provides a good data collapse, as illustrated in Fig. 1d. The data collapse also shows that the scaling function has a broad support, indicating that for a given population size CO_2_ emission can vary over a range of several order of magnitudes. This implies that the impact of population size on CO_2_ emissions is expected to vary from city to city across Europe so that urban scaling only applies on average. This is confirmed by plotting the relation between emission and population size for cities belonging to individual European countries. Estimates of the scaling exponents obtained by fitting separately by country fluctuate considerably (Fig. S2).

To check the robustness of our results, we also consider data from the OpenGHGMap model which reports CO_2_ emissions for 108,000 European cities for the year 2018. Plotting in a double logarithmic scale the emission and population for each city reveals a power law behavior with an exponent $\beta =0.87$ when considering the conditional mean and $\beta _0=1.2$ when considering all the data (Fig. S3).

**Fig. 2:**
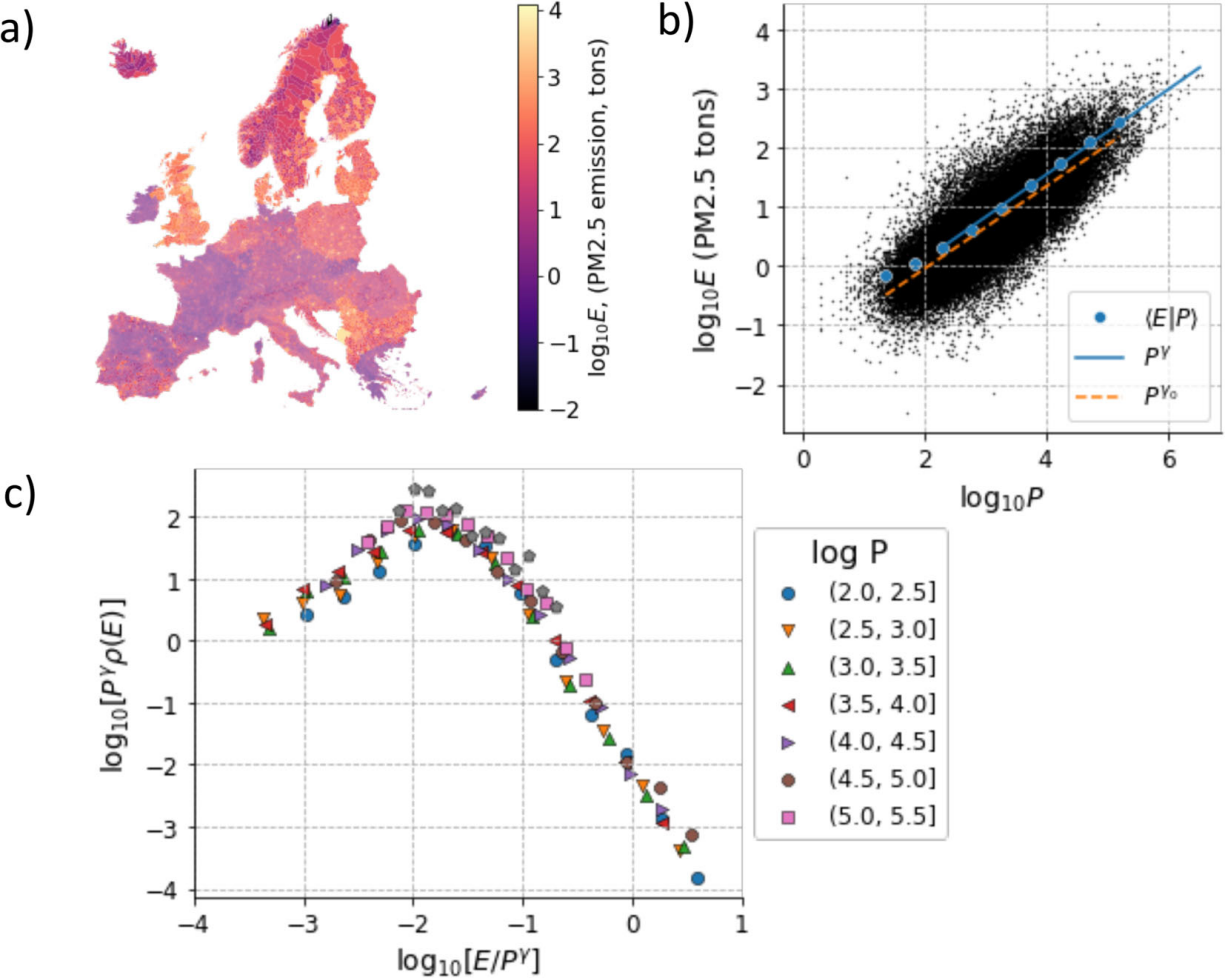
Scaling of PM2.5 emissions with urban population. (a) Map of PM2.5 emissions (as logarithm of the number of tons) of European urban areas. (b) Relation between the logarithms of PM2.5 emissions (*E* PM2.5) and population (*P*), including the expectation value $\langle E|P \rangle$ and the fit with $P^\gamma$, with $\gamma =0.72$. (c) Data collapse of the conditional distributions of PM2.5 emissions at fixed population. Data from GISCO-EUROSTAT and EDGAR (v6.1) for the year 2018

Next, a similar analysis is repeated in the case of PM2.5 emission data from the Edgar database. Also in this case, we aggregate emission data into administrative boundaries and study the dependence of emissions on population sizes. The results are summarized in Fig. 2. The distribution across Europe of PM2.5 is heterogeneous with clear peaks in correspondence to large cities (Fig. 2a), emission and population size are related by a power law with exponent $\gamma =0.72$ that in this case has limited dependence on the way the fit is made, either on the whole data set or on the conditional average (Fig. 2b).

Finally, it is possible to collapse the conditional distributions of PM2.5 emissions at given population size according to the scaling law

3 $$\begin{aligned} \rho (E_{\mathrm {PM2.5}}|P) = P^{-\gamma } \mathcal {G}(E_{\mathrm {PM2.5}} P^{-\gamma }), \end{aligned}$$

where $\mathcal {G}$ is a broad scaling function spanning several decades (Fig. 2c).

### PM2.5 Concentration Is Only Weakly Dependent on Population

**Fig. 3:**
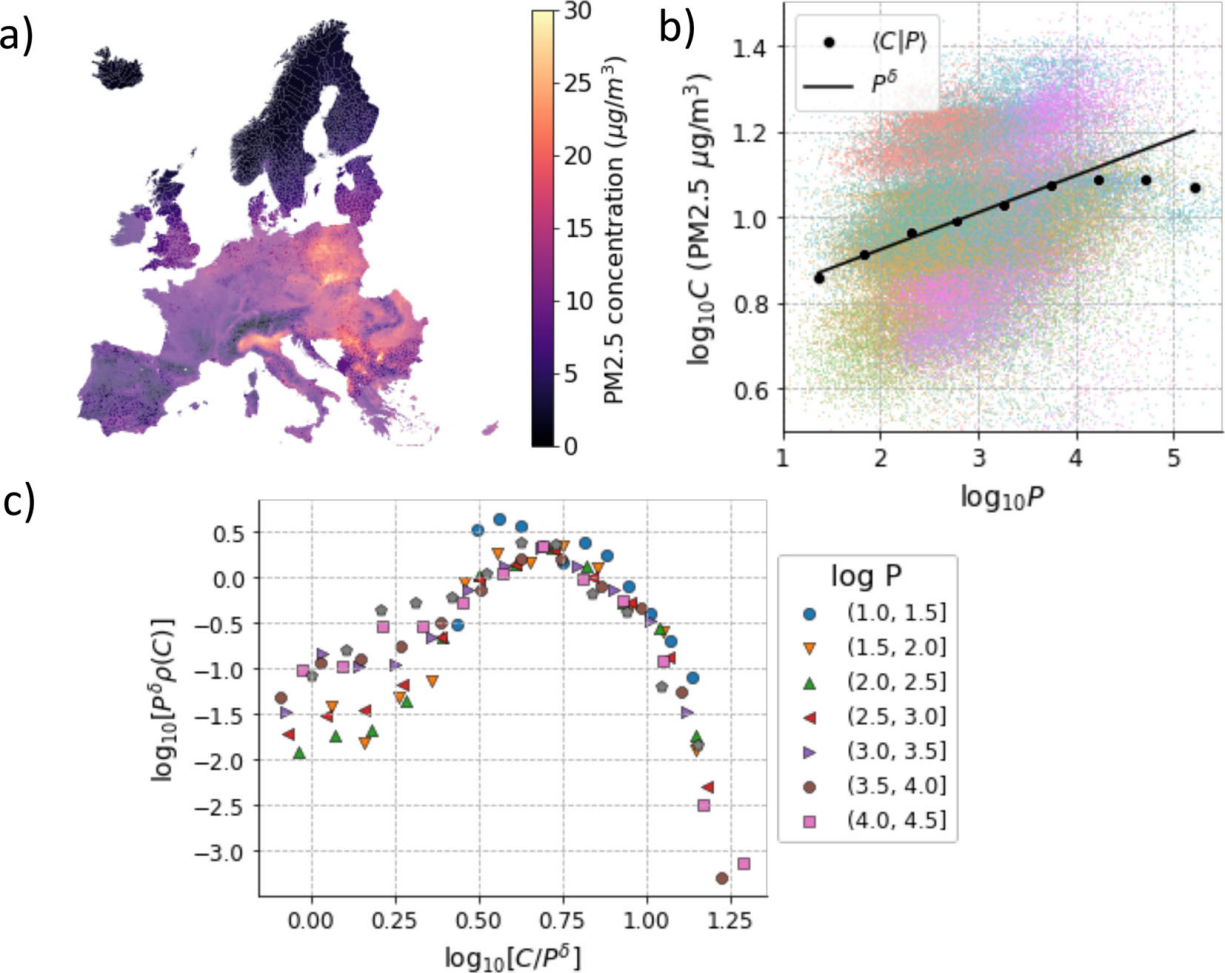
Scaling of PM2.5 concentration with urban population. (a) Map of PM2.5 emissions (as concentration in $\mu$g/m^3^) of European urban areas. (b) Relation between the logarithms of PM2.5 concentration (*C* PM2.5) and population (*P*), including the expectation value $\langle C|P \rangle$ and the fit with $P^\alpha$, with $\alpha =0.08$. Cities are colored according to the country they belong. (c) Data collapse of the conditional distributions of PM2.5 concentrations at fixed population with $\alpha =0.08$. Data from GISCO-EUROSTAT and EEA for the year 2018

Having analyzed PM2.5 emissions in Europe over a year, we also consider PM2.5 concentrations in the air for European cities over the same period. Simple inspection of Fig. 3a shows that while PM2.5 emissions are extremely heterogeneous geographically (Fig. 2a), concentrations are instead varying more smoothly, with extended proximal areas with similar values of the concentration. When we inspect the relation between PM2.5 concentration and population size, we find only a weak interdependence within very large fluctuations among different cities (Fig. 3b). The conditional mean of PM2.5 concentration at fixed population size scales as a power law $\langle C|P \rangle \sim P^\delta$ with a small exponent $\delta =0.08$ only for cities with population less than 10,000 and is independent of population size for larger cities (Fig. 3b). For small cities ($P<10,000$), it is still possible to collapse the conditional distributions using

4 $$\begin{aligned} \rho (C|P) = P^{-\delta } \mathcal {H}(C P^{-\delta }), \end{aligned}$$

with $\delta =0.08$. The variations in concentration among cities with the same population are dependent on the country and so does the fitted exponent (Fig. S4). A summary of the estimates of the estimated values of the exponents obtained from conditional expectation values and from all the data (i.e., $\beta$, $\beta _0$, $\gamma$, $\gamma _0$, $\delta$ and $\delta _0$) is reported in Fig. S5.

### Urban Emissions Are Multifractal While Pollution Is Not

**Fig. 4:**
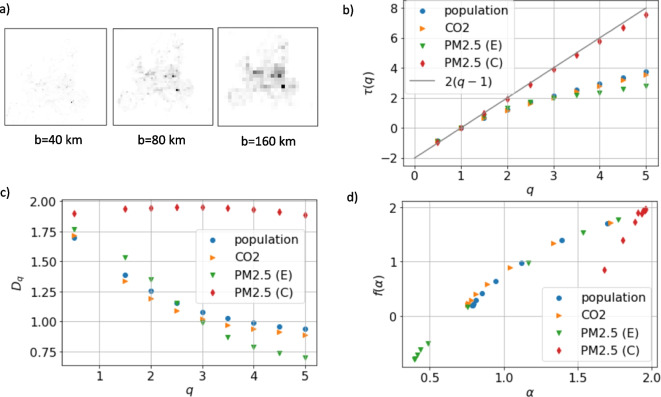
Population and emission distributions are multifractal. (a) Illustration of the box counting method in which a measure, in this case PM2.5 emission, is aggregated at different scales. (b) Multifractal scaling exponents $\tau (q)$ for population, CO_2_, PM2.5 emission and concentration. The prediction for non-fractal exponents $\tau (q)=2(q-1)$ is reported for reference and agrees well with the exponents for PM2.5 concentrations. (c) The corresponding Renyi dimensions $D_q$. (d) The multifractal spectra for population, CO_2_, PM2.5 emission and concentration. Data from GISCO-EUROSTAT, EDGAR (V8.0 and v6.1) and EEA for the year 2018

Given the observed geographical variability in the urban emission and pollution scaling laws, we perform a multifractal analysis [30] of population *P*, CO_2_ and PM2.5 emission ($E_{\textrm{CO2}}$ and $E_{\mathrm {PM2.5}}$), and PM2.5 concentration *C* by aggregating them over grids of variable sizes *b* (Fig. 4a) and then computing the partition function as discussed in the method section. In all cases considered, the partition function scales as a power law with the cell size, $Z_q(b) \sim b^{\tau (q)}$ (Fig. S7), defining a set of exponents for the moments $\tau (q)$, reported in Fig. 4b. For non-multifractal measures on a regular fractal support, the moments exponents should scale as $\tau (q)=D(q-1)$ where *D* is fractal dimension of the support, while for a multifractal measure $\tau (q)$ is a non-linear function of *q*. The moment exponents for the concentration of PM2.5 follows closely the line $\tau (q)=2(q-1)$ which indicates that this measure is not multifractal and has a compact support ($D=2$). On the other hand, all the other measures reveal multifractal scaling since they deviate from a straight line. As a consequence of this, the Renyi dimension, defined as $D_q = \tau (q)/(q-1)$ and reported in Fig. 4c, varies with *q*, with the exception of the one associated with the PM2.5 concentration that is approximately constant.

A non-linear *q* dependence of $\tau (q)$ indicates that there is a spectrum of scaling exponents over the study area or in other words that $\mu _i(b) \sim b^\alpha _i$ for $b\rightarrow 0$, where $\alpha _i$ depends on the cell location. The multifractal spectrum ($\alpha$, $f(\alpha )$), estimated as described in the method section, is reported in Fig. 4d. The function $f(\alpha )$ is the fractal dimension of the set described by the scaling exponent $\alpha$.

**Fig. 5:**
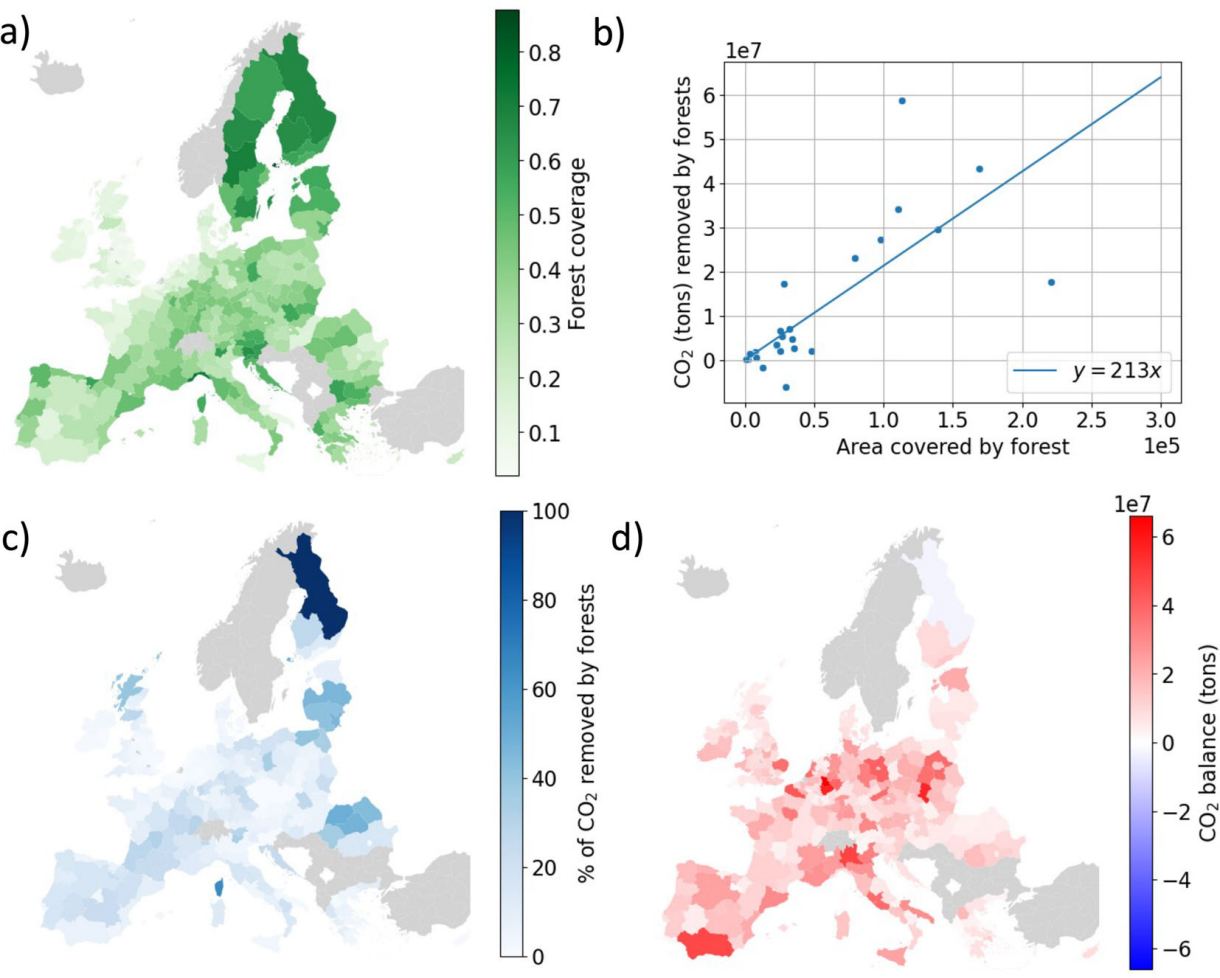
The role of forests in offsetting CO_2_ emissions. (a) The fraction of land covered by forest at the regional level (NUTS2). (b) Quantity of CO_2_ removed from the atmosphere by forests per country. (c) Fraction of CO_2_ emitted removed by forests for each region. (d) Balance of CO_2_ for each region, considering the differences between total emissions and removal from forests. Data from EUROSTAT land cover and the annual European Union greenhouse gas inventory [29]

### Emission and Pollution in Major European Cities

Given that urban scaling laws are geographically dependent, it is interesting to investigate the relations between emission and pollution in major cities across Europe. To this end, we consider here cities with a population size larger than 700,000 and study how CO_2_ and PM2.5 emissions, $E_{\textrm{CO2}}$ and $E_{\mathrm {PM2.5}}$ are related. To correct for the observed population scaling, we normalize the emission variables according to the mean population scaling, using the rescaled variables $\tilde{E}_c=E_{\textrm{CO2}}/P^\beta$ and $\tilde{E}_p=E_{\mathrm {PM2.5}}/P^\gamma$. The two rescaled variable are not significantly correlated (Persson correlation coefficient $r=0.25$, $p=0.2$, see Fig. S8a). We next consider the correlation between PM2.5 emission $E_{\mathrm {PM2.5}}$ and PM2.5 air concentration *C*. In this case, the concentration of PM2.5 is not rescaled since its average value does not depend on the population for large cities (Fig. 3). In this case, we observe a significant correlation between $\tilde{E}_p$ and *C* ($r=0.49$, $p=0.016$). As shown in Fig. S8b, several cities depart from this correlation: cities in northern Italy, like Milan and Turin, have relatively low PM2.5 emission but large PM2.5 concentrations, while Stockholm displays relatively large PM2.5 emissions but low PM2.5 concentrations are recorded. One can explain this observation by geographical consideration: when comparing the map of air concentration with the corresponding elevation map, we see that regions enclosed by mountains such as northern Italy or southern Poland, display very high PM2.5 concentrations (Fig. S9).

**Fig. 6:**
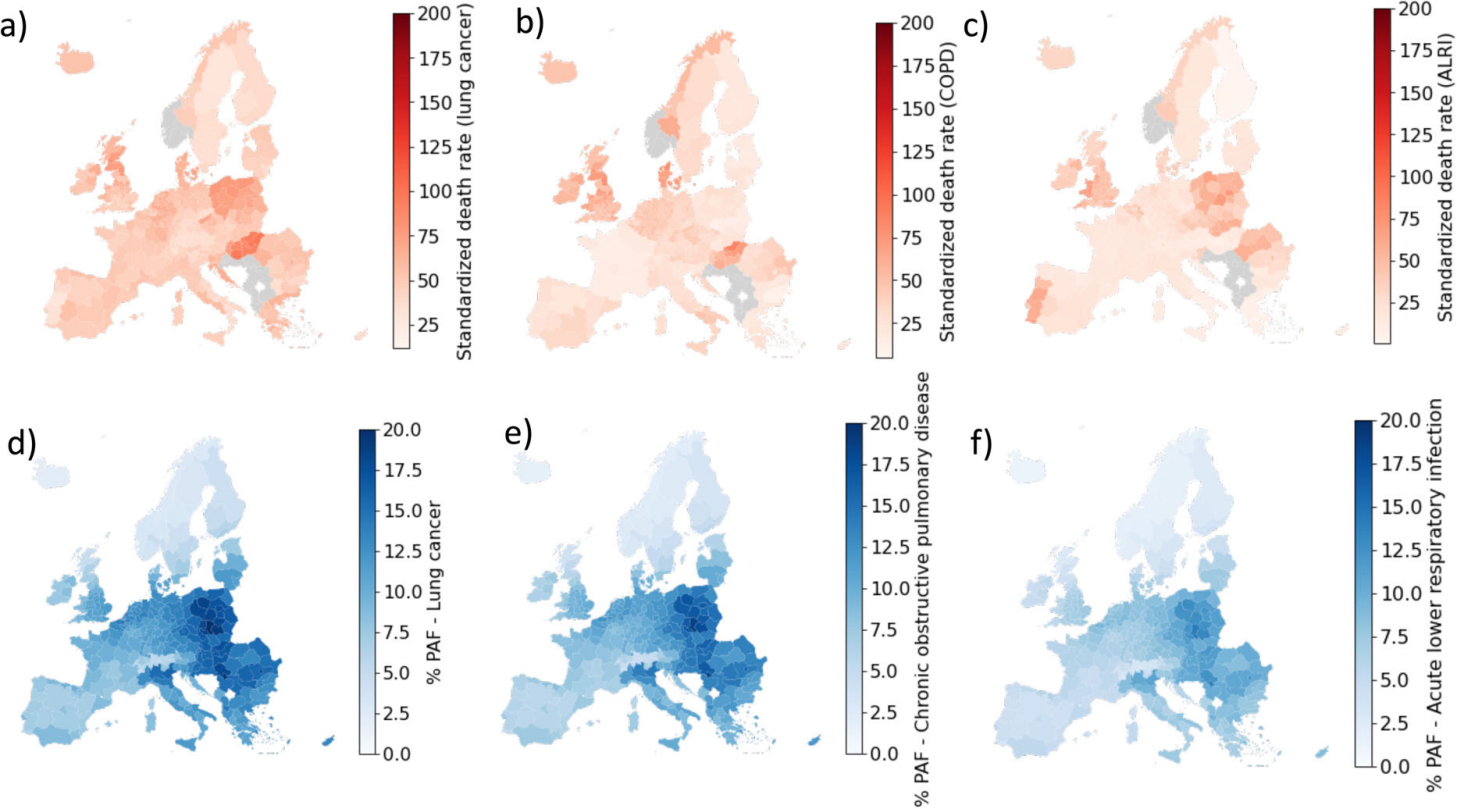
Impact of respiratory diseases in Europe. Map of standardized death rate for (a) lung cancer, (b) acute lower respiratory infections, and (c) chronic obstructive pulmonary disease. Map of population attributable fraction (PAF) to PM2.5 exposure for (d) lung cancer, (e) acute lower respiratory infections, and (f) chronic obstructive. Data from EUROSTAT causes of death database

### Role of Forests in Offsetting CO_2_ Emissions

The values of CO_2_ emissions that we have analyzed do not account for the capture of CO_2_ from vegetation and forests. About 30% of European land is covered by forest which thus contribute to offset part of the CO_2_ that is emitted. The distribution of forest land is heterogeneous as it is illustrated in Fig. 5a showing the fraction of land covered by forests at regional level, according to the NUTS2 classification. According to the Annual European Union greenhouse gas inventory 1990-2019 [29] in 2018, European forests (including the UK) were estimated to capture a total of 285 million tons of CO_2_ representing less than 7% of the CO_2_ emitted in the same year in Europe.

Since forest coverage is not uniform throughout Europe, we analyze the amount of CO_2_ captured by forests at the country level and find that not surprisingly this value is proportional to the forest area in each country (Fig. 5b). We then estimate the fraction of CO_2_ emission that is offset by forests at regional level (Fig. 5c). In most regions, the fraction is less than 10% and it is larger only in a few lowly populated areas. We also compute the CO_2_ balance in each region, defined as the difference between emitted and captured CO_2_ (Fig. 5d). In all regions with the exception of northern Finland, the balance is positive with more CO_2_ emitted than captured. Finally, we also investigate if increasing forest land could have a significant impact in offsetting CO_2_ emissions. According to our estimates, even if the entire European land area would be covered by forest, only 30% of the emitted CO_2_ would be captured.

### Health Impact of PM2.5 Pollution in European Cities

Since long-term exposure to PM2.5 is a known risk factor for several respiratory diseases, we analyzed the mortality due to lung cancer, chronic obstructive pulmonary diseases (COPD) acute lower respiratory infections (ALRI) across Europe at the regional (NUTS2) level (Fig. 6a, b, c). We then used data on PM2.5 concentration to estimate the population attributable fraction (PAF) to PM2.5 exposure or each of these diseases at regional level (Fig. 6a, b, c). The estimate was performed assuming that a long-term concentration of PM2.5 over the year which is equal to the concentration measured in 2018. In reality, however, the PM2.5 concentration decreased in most countries over the past 20 years (Fig. S10a) and therefore the estimated PAF should be considered as a lower bound. The reduction of the PM2.5 concentration in the air across Europe, as well as another important risk factor such as smoking which is also decreasing (Fig. S10b), is likely to be responsible for an observed general decrease in lung cancer mortality (Fig. S10c).

## Discussion

There is currently a global discussion on how to create sustainable cities to address pressing challenges associated with urbanization in its effect on pollution, emissions and health [2]. The increasing concentration of the human population in large urban areas is believed to have a profound impact on CO_2_ and particulate matter emissions, contributing significantly climate change and pollution [32]. An insightful metaphor to summarize the outcome of all the technical and socio-economic processes taking place in cities is the concept of urban metabolism [10]: Cities resemble biological organism as they consume resources and produce waste in a way that is dependent, often in a non-trivial manner, on their scale. The relationship between population, emission and pollution has been investigated widely in the past two decades, leading often to conflicting results [11–16].

In this paper, we contribute to this discussion by considering scaling laws in European cities and focusing our attention on the fluctuations around the mean. To this end, we introduce urban scaling functions describing the conditional distributions of emissions and pollution for cities of comparable population. Using urban scaling functions, we can reinterpret urban scaling behavior showing that the conditional distributions can all be collapsed into a single master curve when variables are properly scaled. Our analysis also reveals large geographical variations in the emission scaling that is captured by a multifractal analysis. In a multifractal, the singularity spectrum changes in different location, consistent with the observed variability in the exponents.

Given that there is a scaling relation between the distribution of emission and population, the multifractality of the emissions might be a direct consequences of the multifractality of the population. In particular if the variable *x* is multifractal and $y=x^a$, then y would also be multifractal with $D_y(q) = a D_x(q)$. In the present case, the situation is slightly more complex since the scaling between population and emission is found for conditional distributions rather than for the variable themselves and this relation between $D_y(q)$ and $D_x(q)$ does not hold. The multifractal nature of emissions and population could also underlie the discrepancies between the exponents measured using conditional expectations and those evaluated using the full data set, since the least-square fit over all the data involves combinations of different moments of the variables.

The use of urban scaling functions changes our perspective on urban scaling since it allows to better qualify the relation between population size, emission and pollution. Once population size effects are discounted by a proper scaling factor, we are able to show that even for rescaled quantities, the relation between pollution and emissons is subject to large geographical fluctuations. For example, we observe that cities in northern Italy produce a moderate amount of relative emissions, but air pollution is very high, while conversely Stockholm has little pollution but relatively higher emissions. This behavior can be explained by geographical and geophysical considerations: northern Italy is an extended shallow valley enclosed by mountains. This leads to frequent air stagnation and emitted pollutants are not dispersed in the atmosphere but increase their concentration in the air [33]. This effect is seen also in other regions enclosed by mountains. Sweden, on the other hand, is a region where air circulation is high and therefore even if emissions are locally large they are efficiently dispersed over an extended area [33].

Common strategies used to mitigate emissions and pollution in cities rely on the implementation of sustainable urban practices, such as the promotion of public transportation, the adoption of renewable energy sources, and improvement in green infrastructure. Trees and forests are known to play a role in mitigating the effect of greenhouse gases emission thanks to their carbon capture capacity [34]. Data show that the carbon capture effect of forest is able to offset only a relatively small fraction of the CO_2_ currently emitted in Europe. Even a significant increase of forest land would still not be able to balance current emissions. Hence, while planting more trees could have various benefits in terms of urban microclimate [35], it does not represent an effective strategy to significantly reduce greenhouse gases in the atmosphere. To this end, one should instead focus on reducing the emissions through the use of more sustainable energy sources.

Finally, we consider the effect on health of urbanization by considering the impact of PM2.5 pollution health. PM2.5 poses significant health risks since these particles are fine enough to be inhaled deep into the lungs and even enter the bloodstream, contributing to respiratory issues such as asthma, bronchitis, and lung cancer. This problem is particularly concerning for vulnerable populations, such as children and the elderly, since long-term exposure to PM2.5 is associated to a higher risk of premature death [36]. With our analysis we estimated the impact of PM2.5 on respiratory diseases across European cities and compared this result with standardized mortality due to the same illness. We observe a general reduction of PM2.5 exposure across Europe over the years, which goes hand in hand with the measured decline of mortality due to respiratory illnesses. We conclude by noticing that while concentrating population in large urban areas might reduce emission per capita, it could also lead to a higher local pollution which is detrimental for the general health of the population living there. The challenge is thus to balance positive global effects for the environment, preserving population health at the local level.

### Supplementary Information

Below is the link to the electronic supplementary material.

Supplementary file 1 (pdf 5653 KB)

## Data Availability

All the data used in the present study are accessible as described in the Methods/Data section.
