# Supplementary material for: Urban Scaling Functions: Emission, Pollution and Health
Source: J Urban Health. 2024 Jul 12;101(4):752–63. doi: 10.1007/s11524-024-00888-2 (PMC11329451; doi:10.1007/s11524-024-00888-2)
Supplement: Supplementary file 1 — (pdf 5653 KB) [file 11524_2024_888_MOESM1_ESM.pdf]

Supplementary Material

Click here to access/download;Supplementary Material;SI.pdf

Supplementary figures

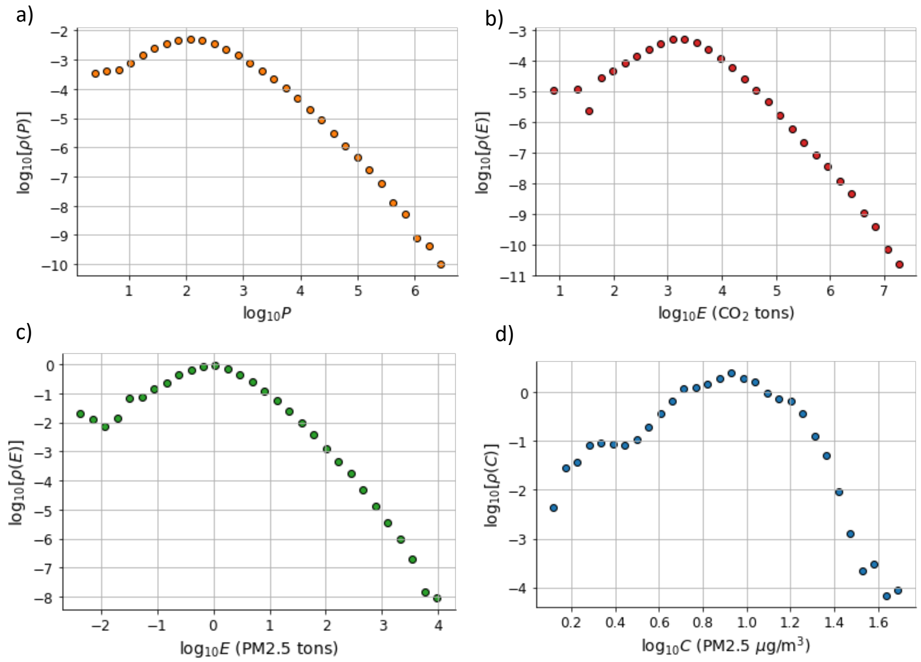

Figure S1: Distribion of a) population, b) CO_2 emission, c) PM2.5 emissions and d) PM2.5 for cities in Europe. Data relate to the year 2018 from a) GISCO Eurostat, b) EDGAR (v8.0), c) EDGAR (v6.1) and d) EEA. Data from GISCO-EUROSTAT and EDGAR (v8.0) for the year 2018.

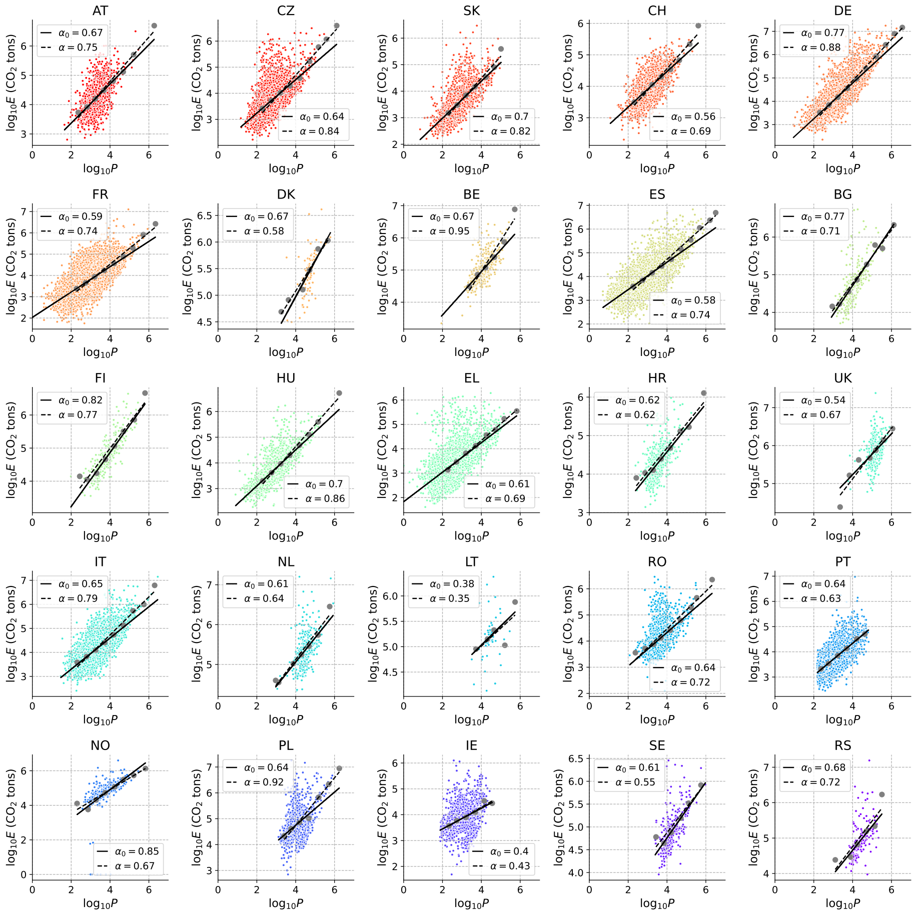

Figure S2: Relation between CO_2 emissions and population size in a double logarithmic scale for cities subdivided by country. Data from GISCO-EUROSTAT and EDGAR (v8.0) for the year 2018.

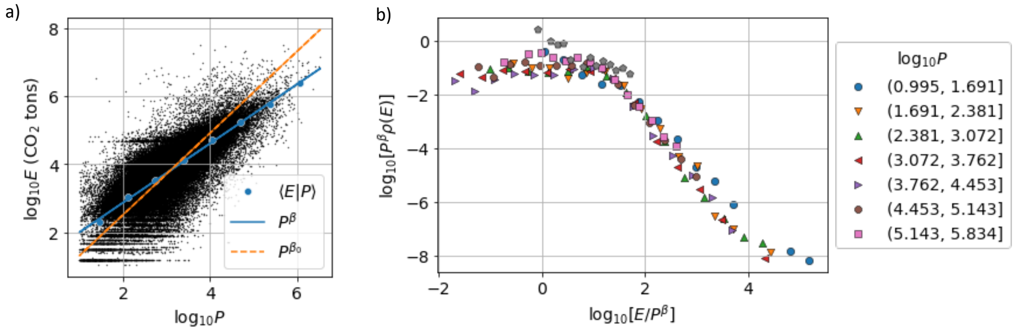

Figure S3: Scaling of CO_2 emission with urban population using the OpenGHGMap data. a) Map of CO_2 emissions (as logarithm of the number of tons) of European urban areas. b) Relation between the logarithms of CO_2 emissions (E CO_2) and population (P), including the expectation value $\langle E|P \rangle$ and the fit with P^{β}, with $\beta = 0.87$. d) Data collapse of the conditional distributions of CO_2 emissions at fixed population.

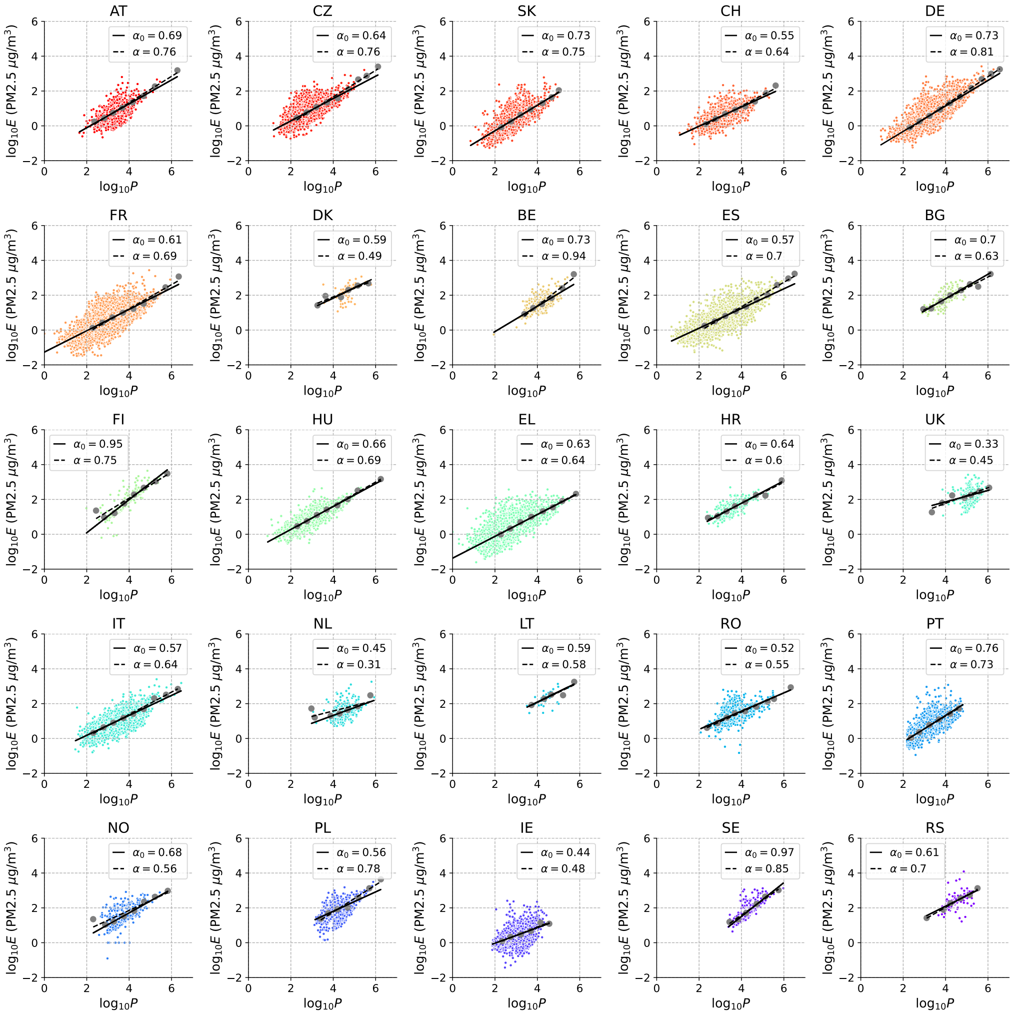

Figure S4: Relation between PM2.5 emissions and population size in a double logarithmic scale for cities subdivided by country. Data from GISCO-EUROSTAT and EDGAR (v6.1) for the year 2018.

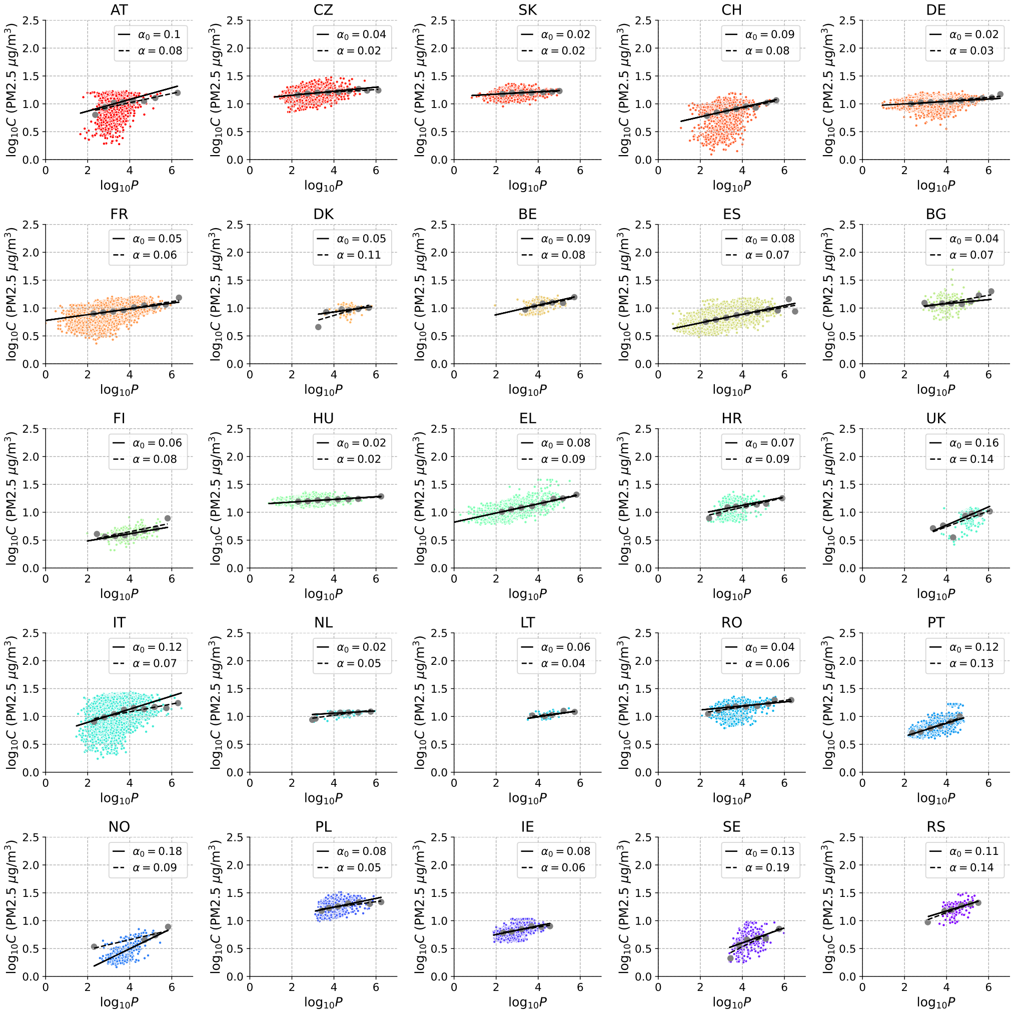

Figure S5: Relation between PM2.5 concentration and population size in a double logarithmic scale for cities subdivided by country. Data from GISCO-EUROSTAT and EEA for the year 2018.

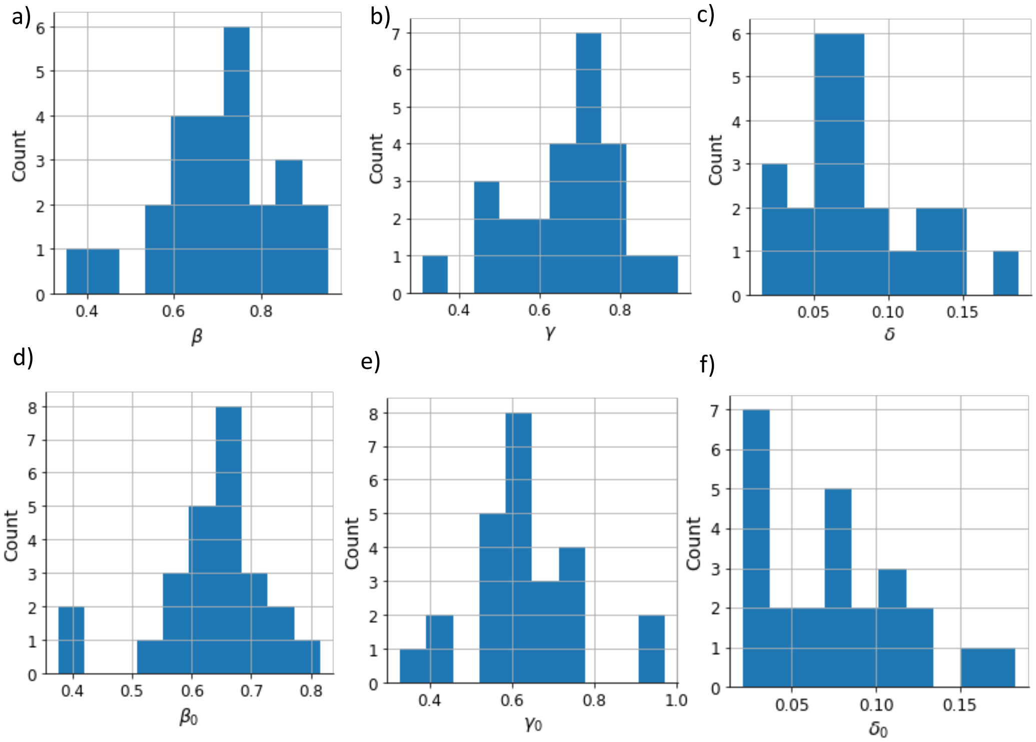

Figure S6: Histograms of the scaling exponents measured in different countries for a) CO_2 emissions, b) PM2.5 emissions and c) PM2.5 concentrations.

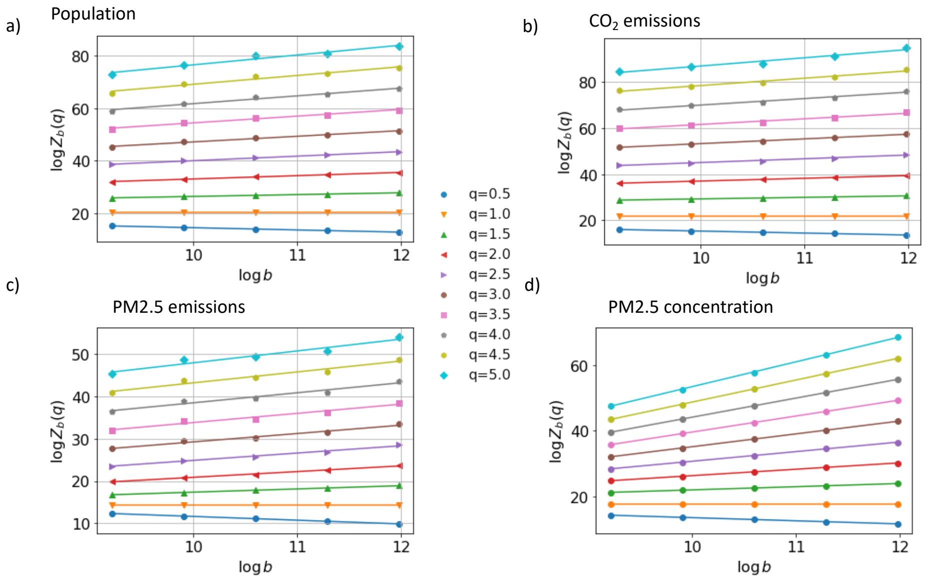

Figure S7: Scaling of the partition function with the cell size for different moments for a) population, b) CO_2 emission, c) PM2.5 emission and d) PM2.5 concentration.

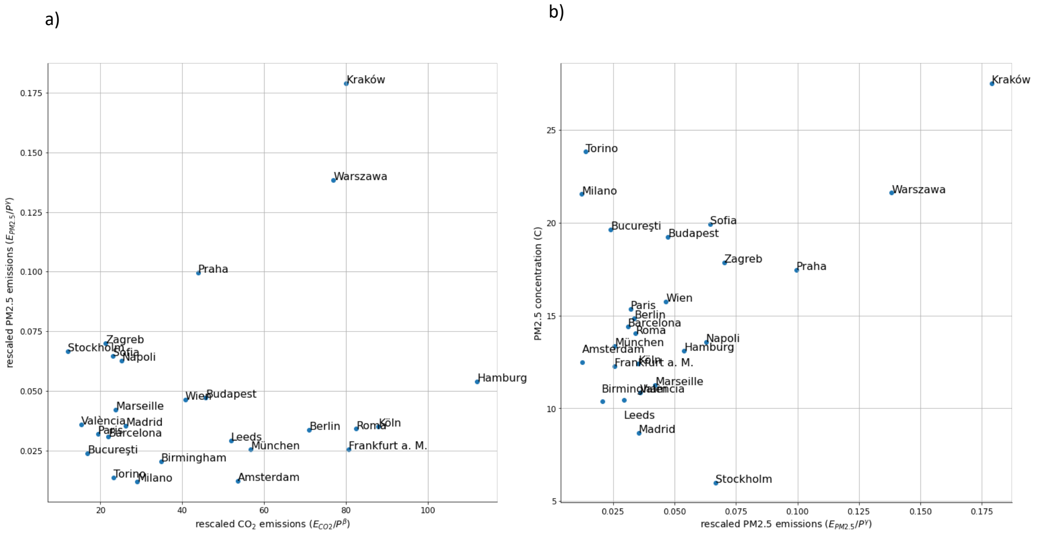

Figure S8: a) Relation between rescaled CO_2 and PM2.5 emissions for major cities. b)Relation between rescaled PM2.5 emission and PM2.5 concentration for major cities. Data from GISCO-EUROSTAT, EDGAR (v6.1 and v8.0) and EEA for the year 2018.

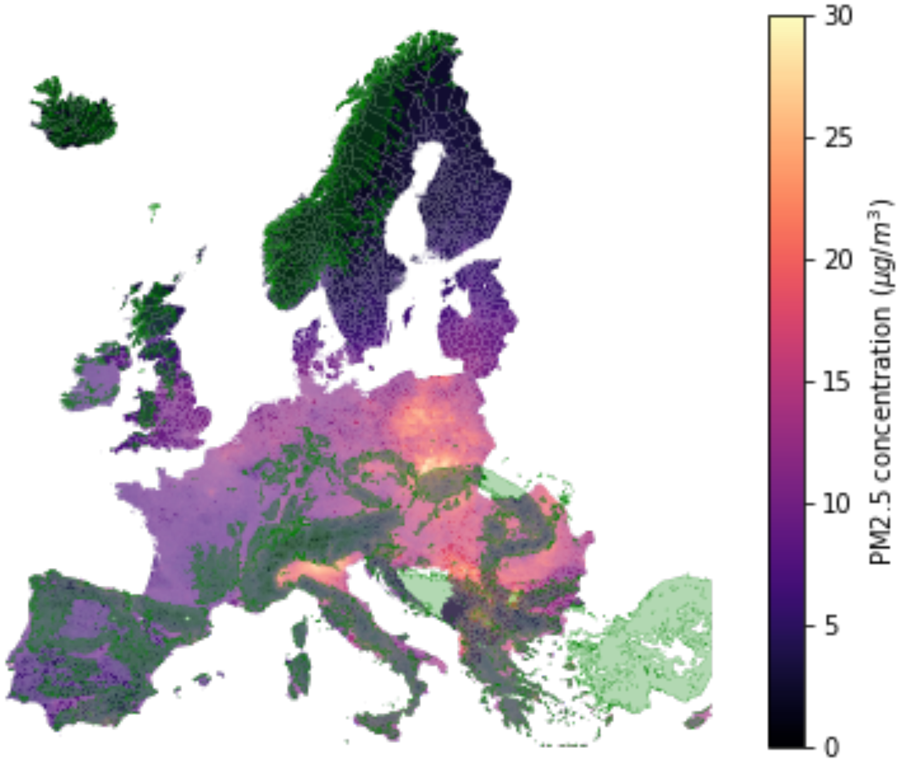

Figure S9: The map of PM2.5 concentration superimposed to the map of European mountains (in green). Data from EEA.

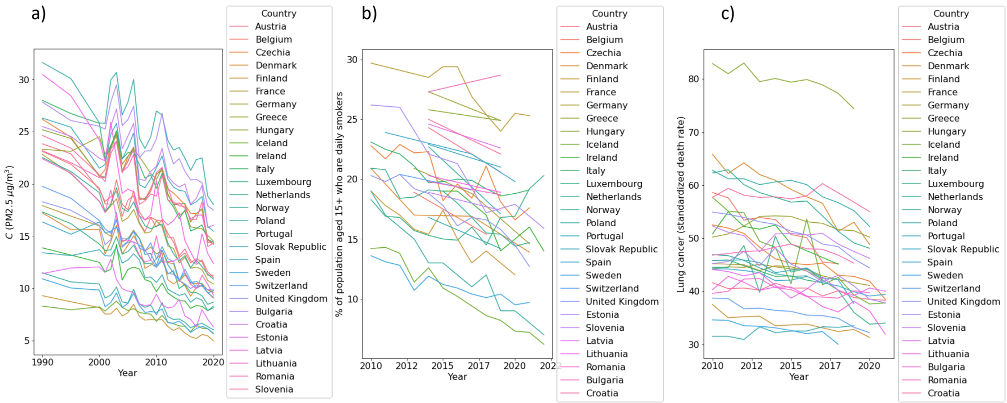

Figure S10: a) The mean concentration of PM2.5 in European countries from 1990 to 2020. b) The percentage of smokers in the population in European countries from 2010 to 2022. c) The standardized death rate from lung cancer in European countries from 2010 to 2022. Data from EUROSTAT.
